# FAST: towards safe and effective subcutaneous immunotherapy of persistent life-threatening food allergies

**DOI:** 10.1186/2045-7022-2-5

**Published:** 2012-03-09

**Authors:** Laurian Zuidmeer-Jongejan, Montserrat Fernandez-Rivas, Lars K Poulsen, Angela Neubauer, Juan Asturias, Lars Blom, Joyce Boye, Carsten Bindslev-Jensen, Michael Clausen, Rosa Ferrara, Paula Garosi, Hans Huber, Bettina M Jensen, Stef Koppelman, Marek L Kowalski, Anna Lewandowska-Polak, Birgit Linhart, Bernard Maillere, Adriano Mari, Alberto Martinez, Clare EN Mills, Claudio Nicoletti, Dirk-Jan Opstelten, Nikos G Papadopoulos, Antonio Portoles, Neil Rigby, Enrico Scala, Heidi J Schnoor, Sigurveig T Sigurdardottir, George Stavroulakis, Frank Stolz, Ines Swoboda, Rudolf Valenta, Rob van den Hout, Serge A Versteeg, Marianne Witten, Ronald van Ree

**Affiliations:** 1 Department of Experimental Immunology, Academic Medical Center, Amsterdam, The Netherlands; 2 Hospital Clinico San Carlos, Facultad de Medicina-UCM, IdISSC, Madrid, Spain; 3 Allergy Clinic, Copenhagen University Hospital, Gentofte, Denmark; 4 Biomay AG, Vienna, Austria; 5 BIAL Aristegui, Bilbao, Spain; 6 Food Research and Development Centre of Agriculture and Agri-Food Canada, St. Hyacinthe, Quebec, Canada; 7 Odense University Hospital, Odense, Denmark; 8 Landspitali University Hospital Reykjavik, Reykjavik, Iceland; 9 Center for Molecular Allergology, Istituto Dermopatico dell'Immacolata, Rome, Italy; 10 Institute of Food Research, Norwich, UK; 11 HAL Allergy BV, Haarlem, The Netherlands; 12 Medical University of Lodz, Lodz, Poland; 13 Medical University of Vienna, Vienna, Austria; 14 CEA, Institute of Biology Technologies, Paris, France; 15 School of Translational Medicine, Manchester Academic Health Science Centre, Manchester Interdisciplinary Biocentre, University of Manchester, Manchester, UK; 16 National Kapodistrian University of Athens, Athens, Greece; 17 Department of Otorhinolaryngology, Academic Medical Center, Amsterdam, The Netherlands; 18 Department of Experimental Immunology, Academic Medical Center, Meibergdreef 9 1105, AZ, Amsterdam, The Netherlands

**Keywords:** FAST, Food allergy, Specific immunotherapy, Subcutaneous, Sublingual, Fish, Peach, Hypoallergens

## Abstract

The FAST project (Food Allergy Specific Immunotherapy) aims at the development of safe and effective treatment of food allergies, targeting prevalent, persistent and severe allergy to fish and peach. Classical allergen-specific immunotherapy (SIT), using subcutaneous injections with aqueous food extracts may be effective but has proven to be accompanied by too many anaphylactic side-effects. FAST aims to develop a safe alternative by replacing food extracts with hypoallergenic recombinant major allergens as the active ingredients of SIT. Both severe fish and peach allergy are caused by a single major allergen, parvalbumin (Cyp c 1) and lipid transfer protein (Pru p 3), respectively. Two approaches are being evaluated for achieving hypoallergenicity, i.e. site-directed mutagenesis and chemical modification. The most promising hypoallergens will be produced under GMP conditions. After pre-clinical testing (toxicology testing and efficacy in mouse models), SCIT with alum-absorbed hypoallergens will be evaluated in phase I/II^a ^and II^b ^randomized double-blind placebo-controlled (DBPC) clinical trials, with the DBPC food challenge as primary read-out. To understand the underlying immune mechanisms in depth serological and cellular immune analyses will be performed, allowing identification of novel biomarkers for monitoring treatment efficacy. FAST aims at improving the quality of life of food allergic patients by providing a safe and effective treatment that will significantly lower their threshold for fish or peach intake, thereby decreasing their anxiety and dependence on rescue medication.

## Introduction

Although reliable figures are still largely unavailable, IgE-mediated food hypersensitivity (hereafter referred to as food allergy) is thought to affect around 1-2% of adults and 4-8% of children, i.e. roughly around 10 million EU inhabitants (reviewed in [1,2]). Recent studies within the FP6-funded project EuroPrevall [3] showed tree nuts (hazelnut and walnut), fruits (apple, peach and kiwi) and peanut are the most common plant foods causing food allergy, followed by vegetables like carrot, tomato and celery. After milk and egg, fish and shrimp are most frequently causing food allergy to animal-derived foods (pers. comm. M. Fernandez-Rivas).

The clinical presentation of food allergy varies from mild local symptoms of the oral cavity, usually referred to as the oral allergy syndrome (OAS), to severe systemic reactions which can include life-threatening anaphylaxis. In the U.S., food-induced anaphylaxis is estimated to cause about 120,000 emergency room visits and 3000 hospitalizations each year [4].

The only available treatment for food allergy is avoidance, in conjunction with rescue medication in case of accidental exposure. However, hidden allergens in composite foods, unwanted contaminations and occasional poor adherence to dietary restrictions make avoidance difficult and ineffective. Therefore there is an urgent need to develop a treatment for food allergy that lowers the threshold significantly and makes avoidance less stringent. Allergen-specific immunotherapy (SIT) is the only treatment available that targets the immunological cause of the disease. It has proven successful in treatment of insect venom allergies and for respiratory allergies such as rhino-conjunctivitis and asthma to pollen and house dust mite [5-7], but due to the duration and invasiveness (i.e. 3-5 years of monthly subcutaneous injections) and the risk of anaphylactic side-effects, SIT is a niche treatment compared to symptomatic drugs, though new alternative routes have been recently successfully explored [8].

Over the past decades, major inhalant and food allergens have been identified, purified, cloned and produced as recombinant proteins. The use of recombinant allergens to replace biological extracts will contribute to enhance the efficacy of SIT by better control over the dosage and elimination of some of the disadvantages (variability in product quality, difficulty in standardization of extracts, sensitization to new allergens) inherent to biological extracts (reviewed in [9]). The first clinical trials using recombinant allergens of birch, grass and ragweed pollen have demonstrated that single recombinant proteins can effectively replace extracts [10,11].

For the development of immunotherapy for food allergy, most attention has so far been given to peanut egg and milk, as these foods are important causes of severe food allergy, mainly in children. Oral immunotherapy approaches for several foods (milk, egg, peanut) show desensitization but no tolerance and commonly have side-effects (well-reviewed in [8]). As children with transient milk or egg allergy seem to have IgE primarily directed to conformational epitopes, sensitive to heat or processing [12,13], two clinical trials focused on investigating tolerance to heated milk and egg products in this population [14,15]. Preliminary studies suggested accelerated tolerance induction, so a follow-up study is ongoing. Subcutaneous allergen-specific immunotherapy (SCIT) as a treatment for peanut allergy has been evaluated using aqueous peanut extract. Although a significant level of efficacy was demonstrated, anaphylactic side-effects, caused by IgE-binding to the injected allergen, were too frequent and the project was abandoned [16,17]. In recent years, sublingual therapy has gained a considerable share of the market for the treatment of respiratory allergies in the form of extract-based drops or tablets. Side-effects are reported to be minimal and efficacy has been demonstrated. The first reports of SLIT with food allergens, date from 2003, in kiwi [18,19]. More recently, SLIT using hazelnut [20,21] and peanut extract [22] has been reported for the treatment of hazelnut and peanut allergy and peach peel extract enriched for LTP was used in a SLIT trial to treat peach allergy [23,24]. These treatments resulted in a significant but moderately increased tolerated dose and systemic side-effects have so far rarely been reported. Despite these quite promising results, in FAST we have decided to target SCIT, the main reason for this being the expected higher efficacy and better compliance and safety, facilitated by performing treatment in an outpatient clinical environment.

To increase safety and develop effective SCIT for the treatment of food allergy, the allergen can be modified in such way that it exhibits significantly decreased IgE-binding potency, i.e. that it becomes hypoallergenic, but retains T-cell reactivity. In addition, these hypoallergens can be absorbed to aluminium hydroxide, which increases safety due to its depot effect and furthermore increases efficacy by its adjuvant effect.

There is still some disagreement concerning the immunological basis of the beneficial effect of immunotherapy. Allergic patients can typically be distinguished from healthy subjects by the presence of allergen-specific IgE antibodies, but a (usually not observed) decrease in specific IgE can not explain the beneficial effect of SIT. The current knowledge on the characteristics of the allergic immune response and its modulation by SIT has developed dramatically beyond the level of serum IgE antibodies, in particular knowledge on other isotypes such as IgG_4 _and IgA, on various subsets of helper T-cells (Th-cells) and on the role of innate antigen presenting cells (like dendritic cells (DCs). Essentially there are two extremes for explaining the beneficial effect of SIT: inhibition of allergic reactions by blocking IgG_4 _and IgA antibodies or by a shift from Th2 to Th1/Treg. FAST aims at induction of both using hypoallergenic but immunogenic recombinant allergens. Although double-blind placebo-controlled food challenge (DBPCFC) will always remain the primary read-out for establishing efficacy, it is not an appropriate tool to use at many (early) time-points during treatment. However, reliable early (composite) biomarkers for efficacy that correlate with the outcome of the DBPCFC are not (yet) available. To improve and identify relevant (composite) biomarkers for monitoring efficacy of immunotherapy it is important to further unravel the mechanism of protection in patients responding favorably to immunotherapy. In depth monitoring of humoral and cellular immune parameters will help identify such (early) biomarkers for efficacy.

Within this context it is the objective of the EU-funded collaborative project FAST to develop a safe and effective immunotherapy against persistent and life-threatening food allergies. The project includes 15 partners from 11 different countries. To address all the main objectives indicated above, the partnership first focuses on producing and testing a number of different mutant (hypo-) allergens (and wild-type allergens for comparison). Additionally, there are three companies, two partners from the pharmaceutical industry (BIAL, Spain and HAL Allergy, The Netherlands) and one biotech company (BIOMAY, Austria) that will focus on the production of the chosen hypoallergens under good manufacturing practice (GMP) for the clinical trials. In the consortium there are six clinical centers participating in six countries, chosen on the basis of expertise and geographic background. Lastly, allergen-specific MHC class II tertramers/multimers will be developed as well as mouse models for immunotherapy with hypoallergens.

## The allergens

Ninety percent of all food allergies are caused by ± 10 foods. Allergy to some of these foods i.e. milk and egg, are outgrown in the vast majority (milk) or up to 50% (egg) of children before the age of five. Although these are certainly relevant, persistent food allergies that stay throughout adulthood perhaps represent a more important target for developing immunotherapy (IT). Most attention for the development of IT for food allergy has so far been given to peanut and despite being usually outgrown, also to egg and milk. Apart from prevalence, the main reason for this focus is high risk of severe reactions induced by these foods. Allergy to fruits, like peach, and to tree nuts, fish and shrimp are also high on the list of candidates to develop novel therapies: allergy against these foods is prevalent, persistent and potentially life-threatening and avoidance negatively affects a healthy diet. For the development of a new concept for the treatment of severe persistent food allergies based on recombinant hypoallergens, the best prospects for reaching clinical testing at Phase II^b ^level within the life-time of an EU project are treatments targeting food allergies that are dominated by a single major allergen. As described above, in the case of severe allergies to peanut but also to tree nuts there are at least three major allergens, so multiple recombinant allergens would be needed. Therefore, in the FAST project we target two foods, one from animal origin, fish, and one from plant origin, peach. Severe reactions to fish and peach are both linked to a single dominant allergen, parvalbumin (Cyp c 1/Gad c 1) and lipid transfer protein (Pru p 3) respectively. Peach was chosen as a representative of all fruit allergies linked to lipid transfer protein. Both the natural and recombinant wildtype (WT) are compared to hypoallergenic variants produced in FAST.

Two ways of rendering allergens hypoallergenic are used in FAST. Recombinant technology allows modification by site-directed mutagenesis and with this method hypoallergenic mutants have been successfully developed for several food allergens. These include the major peanut allergens Ara h 1, 2 and 3 [25], the major fish allergen Cyp c 1 [26,27] and the major fruit allergen Pru p 3 ([28] and own observation). Another approach is chemical modification. Since the 1970s, allergen extracts are treated with glutaraldehyde resulting in cross-linked proteins with reduced allergenicity, (also known as allergoïds) and hypoallergenicity can also be induced by reduction/alkylation in allergen molecules containing disulfide bridges. Here for the first time, we will apply this concept to recombinant food allergens.

### Fish

**Figure 1:**
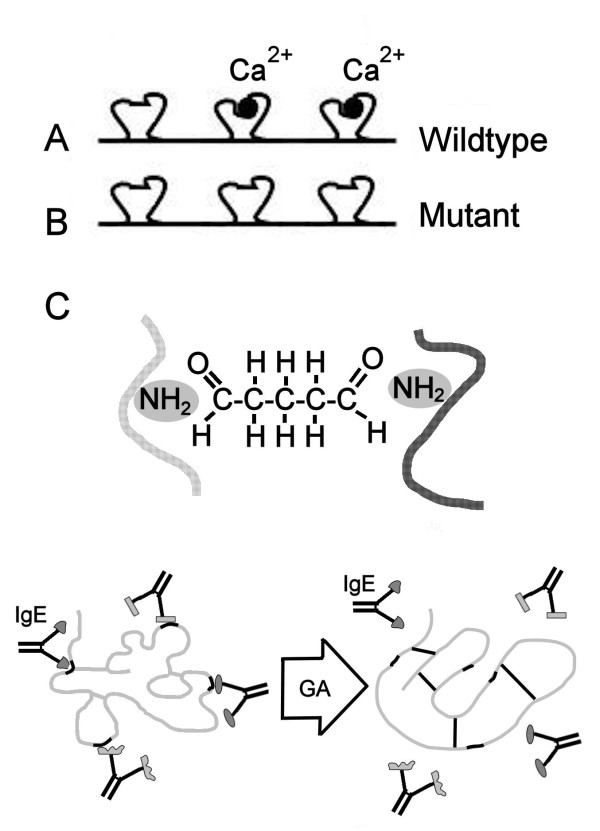
**Four different (hypo-)allergenic constructs are proposed in developing a construct for fish SIT**. A/B: n/r wild-type Cyp c 1 and the Calcium-binding site double mutant as described before [26,27], C: glutaraldehyde treated rCyp c 1 (involves covalent linking of free amino-groups, as shown).

For the development of immunotherapy for fish allergy, we will focus on the major allergen from carp (*Cyprinus carpio*), the parvalbumin (Cyp c 1). Parvalbumins are small, acidic calcium-binding buffer proteins found in fast muscle of lower and higher vertebrates. They have been identified as the major fish allergens [26]. Parvalbumin is a 3 EF-hand calcium-binding protein. It has remarkable stability to heating and digestion, which explains why, despite cooking and exposure to the gastrointestinal tract, it can sensitise patients [29]. A wild-type recombinant (r) and three hypoallergenic mutants representing of Cyp c 1 have been developed by our partner in Vienna [26,27]. Wild-type rCyp c 1 was shown to be highly cross-reactive to other fish parvalbumins like from cod, salmon and tuna, ensuring broad coverage of fish allergies. For many patients IgE binding was Ca^2+^-dependent; mutating the two functional Ca^2+^-binding sites resulted in loss of most of the secondary structure and hypoallergenicity in dot-blot-inhibition (n = 4) and a ~100-fold reduction in biological activity in basophil histamine release (BHR; n = 1) compared to the wild-type recombinant protein. We are producing and purifying the natural and wild-type rCyp c 1, the hypoallergenic double mutant and a chemically modified wild-type rCyp c 1 (so-called allergoïd, produced by glutaraldehyde treatment) for pre-clinical evaluation in FAST (Figure 1).

### Peach

**Figure 2:**
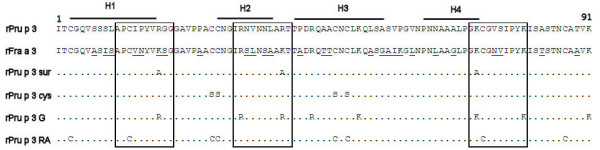
**Seven different (hypo-)allergenic constructs are proposed in developing a construct for peach SIT**. wt and rPru p 3 (sequence shown), a "natural hypoallergenic" rFra a 3 (changes in sequence compared to Pru p 3 are underlined), rPru p 3 sur: a surface mutant (3 amino acids mutated to Alanin), rPru p 3 cys: 4 cysteines mutated to serine, rPru p 3 G: glutaraldehyde treated rPru p 3 (involves free amino-groups, indicated), rPru p 3 RA: reduced and alkylated rPru p 3 (involves all cysteines). The known IgE-binding sites of Pru p 3 are boxed, H1-4 indicate the α-helices.

For peach (*Prunus persica*) allergy, we focus on its major allergen, the non-specific lipid transfer protein (LTP) Pru p 3. LTPs have been identified as the culprit of severe fruit allergy mainly for fruit-allergic patients in Mediterranean countries [30,31]. Sensitization is usually caused by peach LTP and cross-reactivity between highly homologous LTPs results in clustered fruit allergies. 50-95% sequence identity commonly gives rise to IgE cross-reactivity, however, not always leading to clinical fruit allergy. Both apple and strawberry LTP (Mal d 3 and Fra a 3, respectively) are approximately 80% homologous to peach LTP [32], but where apple allergy is common among LTP-sensitized peach allergic patients, strawberry allergy is not. Therefore, Fra a 3 may be a naturally occurring hypoallergen. For Pru p 3, several charged surface-exposed amino acids in three regions across the molecule have been proposed to play a role in IgE binding [33,34]. The structure of LTP is highly dependent on its four disulfide bridges. Mutation of a single cysteine in each pair forming a disulfide bridge has been shown to significantly reduce the allergenicity of the major *Parietaria *weed LTP, Par j 1 [35] and very recently for Pru p 3 [28]. All in all, we are using five different strategies to produce hypoallergenic LTP for safe treatment of fruit allergy; we produce and purify wild-type natural and rPru p 3 and rPru p 3-mutants (surface-exposed amino acids and disulfide bridges), chemically modified wild-type rPru p 3 (reduction/alkylation and glutaraldehyde treatment) and rFra a 3, all tested for hypoallergenicity. These constructs are summarized in Figure 2.

All (hypo-)allergen preparations as described above are physico-chemically characterized by far UV CD-spectroscopy, mass-spectrometry and size-exclusion chromatography/dynamic light scattering. Stability is tested and hypoallergenicity is assessed in patients' sera (n = 30, selected as described below) with ImmunoCAP (CAP)-inhibition and BHR. Furthermore the capacity of wild-type molecules (recombinant and natural), mutants and allergoïds, to stimulate T-cell proliferation (using PBMCs from fish/fruit-allergic patients) and induce IgG antibodies in rabbits and/or mice is assessed.

On the basis of these analyses, using a weight-of-evidence approach, the most appropriate hypoallergenic but still immunogenic molecule is selected for GMP production, toxicity testing and subsequent clinical trials.

## Clinical studies

In the consortium there are six participating clinical centers in six countries: Odense (OUH, Denmark), Łódź (MUL, Poland), Reykjavik (LSH, Iceland), Madrid (HCSC, Spain), Athens (NKUA, Greece) and Rome (IDI, Italy). These centers have been chosen on the basis of their specific expertise in food allergy and DBPCFC and their geographic background.

The methodology will be identical to those of the clinical studies performed within the EU-funded integrated project EuroPrevall [3] that were coordinated by the partner from Madrid (including standardized case record forms (CRFs), methods for skin prick testing (SPT) and double-blind placebo-controlled food challenges (DBPCFCs). From the clinical studies of EuroPrevall, we have concluded that fish allergy is observed across Europe but it is especially frequent in Iceland, Spain, Greece, Italy and Poland. Moreover, the Danish groups have published studies with 30+ codfish allergic patients [36,37]. Peach allergy caused by LTP is frequently found in Spain, Italy and Greece. Together, these centers therefore provide the necessary expertise and cover some of the most important areas for fish and peach allergy in Europe. All clinical studies and clinical trials will be performed with the approval of the local ethics committees, and according to the national and European regulations.

### Allergic patients for evaluation of candidate allergen molecules

Clinical centers will enroll fish (parvalbumin) and/or peach (LTP) allergic patients. The aim is access to biological samples for the evaluation of hypoallergenicity and T-cell reactivity of parvalbumin- and LTP-variants described in the previous section. In addition the patients are screened for MHC II alleles to select appropriate candidates for evaluating the relevance of T-cell epitopes. For both fish and peach we aim at inclusion of 30 patients, evenly spread over the relevant clinical centers, respectively. The sample size has been calculated on the basis of the expected reduction in allergenicity of the candidate molecules.

Patients are recruited at the 6 clinical centers involved in the project. Inclusion is based on age (between 12 and 65) and a convincing clinical history for fish or peach, a positive SPT and DBPCFC with fish or peach, and a positive serum IgE test to rCyp c 1 (in case of fish) or rPru p 3 (in case of peach). Patients with severe anaphylaxis are excluded from DBPCFC to establish their current reactivity to the foods. They will be included in the clinical trials.

### Phase I/II^a ^clinical trials

Phase I/II^a ^clinical trials using hypoallergenic recombinant fish parvalbumin or peach LTP will be carried out in Denmark and Spain, respectively. The main objectives of the Phase I/II^a ^trials will be dose-finding and to assess safety and pharmaco-dynamics. Safety will be assessed with a careful recording of all adverse events and adverse reactions. For pharmaco-dynamics allergen-specific IgE, IgG/G_4_, IgA and T-cell proliferative responses will be monitored. Adult subjects allergic to fish parvalbumin and to peach LTP recruited by the above mentioned clinical partners will be invited to participate. 24 individuals with a proportion active:placebo of 3:1 will be included for DBPCFC. The hypoallergens will be tested in subgroups of patients at different dosing schemes for 3 months in a staggered manner at intervals of 2 weeks to allow for intermittent safety reviews.

### Phase II^b ^clinical trials

If no major side-effects are reported in Phase I/II^a ^trials, Phase II^b ^trials will be performed. Patients will be recruited according to the inclusion limits described above. The studies will be randomized, double-blind placebo controlled (DBPC), with a proportion of active and placebo treatments of 2:1.

The primary outcome of Phase II^b ^trial is the response to DBPCFC performed after the treatment that will be compared to the pretreatment challenge by survival analysis. The estimated total number of patients needed is 105 per trial, 70 active and 35 placebo subjects. For fish this means that 12 active and 6 placebo subjects should be recruited in each clinical center (total number 108), and for fruit the respective figures will be 24 active and 12 placebo per center (total 108). Treatment at maintenance dose will last for 6 months (monthly injections).

Safety and tolerability will be assessed by careful recording of adverse events. Investigators will evaluate the nature and severity of the events, in attempt to determine the causal relationship with the immunotherapy. Registry and classification of adverse events will be performed in accordance with local regulations. The primary outcome of efficacy in the Phase IIb trials will be the response to the intake of fish or peach assessed by a standardized DBPCFC that will be performed before the start of the treatment and at the end of it. DBPCFC performed after the treatment will be compared to the pretreatment challenge by survival analysis. Secondary outcomes of efficacy will be changes in SPT reactivity (to a fish/peach extract), in specific IgE, IgA and IgG_4 _(to the respective purified allergen) in biological activity of IgE (BHR) and in T-cell reactivity.

## Immunology

For adequate monitoring and future improvement of immunotherapy for food allergy, it is necessary to establish which immune mechanisms are protective. First, this will be studied in a mouse model of food allergy immunotherapy. Later, comprehensive immunological studies during Phase II^b ^clinical trials will be carried out.

### Immunotherapy with hypoallergens in a mouse model

A model of food hypersensitivity, first described by Hugh Sampson *et al*. [38] is used to evaluate the potential of hypoallergens to treat mice sensitized with purified nCyp c 1 or nPru p 3. In this model, C3H/HeJ mice are orally sensitized by repeated intra-gastric administration of allergen in combination with cholera toxin as adjuvant, followed by challenge with a single large dose of allergen to provoke an allergic reaction. The characterization of the allergic responses is based on well-established *in vivo *parameters (symptomatic scoring, vascular leakage and immediate type skin tests) and *in vitro *tests (measurement of allergen-specific immunoglobulin in serum and mucosal secretions (ELISA), cellular responses in spleen and lymph node cells (T cell proliferation and cytokine secretion assays), plasma histamine levels and histological tests (intestine and lung sections). Pilot experiments using varied feeding protocols and different antigen concentrations are performed to establish an optimal sensitization (characterized by highest production of IgE) and challenge regime. The latter will be important also to elaborate a symptomatic scoring system that can be used to confirm the benefits of the treatments being proposed.

The model will be used to assess both subcutaneous immunotherapy with the selected hypoallergenic parvalbumin and lipid transfer proteins. Apart from evaluation of these candidate molecules for human trials, the mouse model is used to investigate the immune mechanisms of subcutaneous immunotherapy for food allergy. All animal experiments are carried out according to national and European regulations.

### Changes in immune response during immunotherapy

As outlined in the introduction, the immune mechanism of allergen-specific immunotherapy has only been studied in some detail for treatment of respiratory allergies [5,6] and to a lesser extent, bee venom allergy [7]. For the treatment of food allergy, only a limited number of well-designed immunotherapy studies (two SIT studies for peanut [16,17] and two SLIT studies for hazelnut and peach, respectively [20,21,23] have been performed, without detailed mechanistic studies. Immunological investigations were limited to IgE and IgG serology. In this project, we aim to treat with the major active compound only and also monitor serum antibodies to this major allergen. This will establish whether there is a correlation between efficacy and IgG and IgA responses to the active compound.

Additionally we will study what exactly happens to allergen-specific IgE. Rise and fall of IgE-titers and changes in specificity will be studied. Additionally, it is important to investigate what causes the inhibition of early and late-phase reactions in the presence of relatively stable IgE titers; is it qualitative changes in IgE (which will be monitored by measuring biological activity in histamine release tests) or potentially blocking effects of IgG and/or IgA antibodies.

The number of clinical trials where allergen-specific T-cell responses were monitored and characterized in detail is very limited. In order to adequately monitor and improve efficacy of food allergy SCIT, it is essential to acquire in depth knowledge on the mechanism. To establish the role of different Th-cell subsets in the mechanism of protection induced by immunotherapy, blood samples will be obtained prior to, during and after completion of therapy to analyze T-cell proliferation (FACS analysis), cytokine production (Luminex/ELISPOT/ELISA) and surface marker expression (FACS analysis) to determine the phenotype of allergen-specific T-cells. To be able to selectively focus at allergen-specific T-cells, food-allergen-specific tetramer (or ultimer) reagents will also be developed (see below).

With all these studies we can further elucidate the immune mechanism of allergen-specific immunotherapy and hopefully identify useful (early) biomarkers for efficacy.

### T-cell epitopes of parvalbumin and LTP

To be able to study the phenotype of allergen-specific T-cells in healthy and allergic subjects and follow changes in phenotype during SCIT, parvalbumin- and LTP specific MHC class II (MHC II) tetramers will be developed. Essentially, there are two unknowns: 1) which MHC II molecules are most relevant for antigen presentation in case of parvalbumin and LTP 2) which T-cell epitopes play a dominant role in parvalbumin- and LTP-specific T-cell responses? Since the beginning of the FAST project, three papers have addressed the latter question for peach LTP [39-41] all pointing to the regions Pru p 3_12-27 _and Pru p 3_57-80 _as carrying important T-cell epitopes. Immuno-purified MHC II molecules covering a large part of the Caucasian population (twelve different HLA-DR and HLA-DP4 molecules) will be used to perform binding studies with overlapping peptides spanning the sequence of Cyp c 1 and Pru p 3 using the HLA express system [42]. Affinity of binding to MHC II, the capacity to stimulate T-cells, and the prevalence of the particular MHC II molecule among Europeans and their availability for production as tetramers will decide which T-cell epitopes will be custom-ordered. The aim is to have sufficient (HLA-) coverage to be able to study differences between healthy and allergic T-cell responses and changes during immunotherapy. This will provide the tools to identify and characterize parvalbumin- and LTP-specific T-cells at epitope level.

## Concluding remarks

The FAST project will increase our understanding on how to bring the treatment of food allergy to a higher level, i.e. by adding an alternative to the spectrum of treatment modalities for food allergy that is currently restricted to avoidance and rescue medication, and SLIT or OIT. Overall, allergy is recognized as a major disease affecting around 30-40% of the population. Food allergy is estimated to affect about 5% of the population, but additionally it also is a major disease because of its great impact on the quality of life. Food allergy is potentially life-threatening and the risk of accidental intake causes great fear and ultimately leads to social isolation. The FAST project aims at significantly lowering thresholds and consequently improving quality of life of food allergy sufferers.

FAST investigates the development of novel therapies for the treatment of food allergy by combining the principles of traditional allergen-specific immunotherapy with biotechnology (hypoallergenic recombinant allergens). Standardization of allergen extracts has been a major challenge over the past decades. The introduction of highly purified products will end the situation where standardization of variable biological products puts an increasing burden on quality control departments and regulatory authorities.

The research proposed in FAST aims at the development of treatment for diseases that start in early childhood, using top clinical, biotechnological and immunological research. The strategy chosen will not involve children up to the stage of Phase II^b ^clinical trials. Phase I/II^a ^clinical trials will be carried out in adults only. Moreover, children enrolled in Phase II^b ^clinical trials will not be under 12. FAST aims to develop a novel therapy for food allergy that will have a positive impact on the diet of food allergic patients improve their quality of life, allowing them to stop avoiding fish and fruit which are important components of a healthy "non-obese" diet.

Once more the importance of child health is addressed. As mentioned food allergy is a disease that in most cases starts in very young children. The FAST project aims to develop a therapy for food allergy that in the future can also be safely used in children.

Obviously, the approach chosen by FAST is applicable to the treatment of other food but also respiratory allergies. Moreover, successful therapy is the first step towards allergen-specific preventive immunotherapy or vaccination. FAST targets a chronic disease that is potentially life-threatening by anaphylactic shock. Food allergy is currently untreatable, avoidance being the only remedy. It will do so by using the established (subcutaneous) route of administration. It will use established (chemical modification) and emerging (mutants) methods to achieve hypoallergenicity, aiming at increased safety of the treatment. It aims at replacing extracts by highly purified recombinant allergens. This will allow more accurate administration of active ingredients, which will hopefully improve efficacy. The mechanism of action will be investigated with a major focus on the potential role of allergen-specific regulatory T-cell. To be really able to study these cells at the level of specific epitopes, food allergen specific tetramers will be developed. Overall, the project aims at developing a novel strategy to replace avoidance and rescue medications as the only way to treat food allergy: allergen-specific immunotherapy with biotech hypoallergens.

## Competing interests

The authors declare that they have no competing interests. This study was funded by the EU (201871).

## Authors' contributions

LZ and RvR wrote most of the manuscript, and the FAST steering committee (MFR, LP, AN and RvR) did most of the work designing the project, all other authors were involved in the design of the study and have read and approved the manuscript.
